# Duodenal Phytobezoar Treated With Endoscopic Removal: A Case Report

**DOI:** 10.7759/cureus.79147

**Published:** 2025-02-17

**Authors:** James R Conomea, Adam K Bobak, Harthik Kambhampati, John Stauffer, Michael Herman

**Affiliations:** 1 School of Medicine, Lake Erie College of Osteopathic Medicine, Bradenton, USA; 2 Gastroenterology, Borland Groover, Jacksonville, USA

**Keywords:** bezoar, case report, duodenum related cases, gastric outlet obstruction (goo), gastrointestinal (gi), phytobezoar, upper endoscopy

## Abstract

Gastric outlet obstruction (GOO) is a blockage within the proximal gastrointestinal tract that most commonly occurs within the stomach. GOO can present with symptoms like nausea, vomiting, upper abdominal pain, early satiety, weight loss, or abdominal distention with a succussion splash. Diagnosis is most evident with an abdominal X-ray showing dilation proximal to the obstruction and air-fluid levels, but other visualization techniques like abdominal CT and upper endoscopy may be useful in the diagnosis depending on the underlying cause. This clinical condition has multiple causes, with malignancy being the most prominent; however, rare cases like gastrointestinal bezoars may occur in the setting of altered gastric motility. We present a case of a 47-year-old male with GOO caused by a phytobezoar, a mass of undigested vegetable material, in an uncommon location, the duodenum. Upon endoscopic removal of the bezoar, the patient had a resolution of symptoms, and he was instructed to modify his dietary habits, the underlying cause of the obstruction.

## Introduction

Gastric outlet obstruction (GOO) is a gastrointestinal disorder caused by blockage within the stomach, pylorus, or duodenum. Multiple etiologies can result in GOO, including malignancy, peptic ulcer disease (PUD), motility dysfunction, and diabetic gastroparesis [1,2]. Most patients presenting with GOO have a malignancy causing luminal obstruction, such as pancreatic adenocarcinoma or distal gastric cancer [1,3]. Peripancreatic malignancy carries a 15-20% risk of GOO development [4]. Benign etiologies include PUD, inflammatory disorders, and iatrogenic mechanisms [1,5]. PUD is the most frequent etiology of benign pathologies and progresses to GOO in 2-5% of patients; this percentage has dropped due to the successful treatment of *H. pylori* infections [1,4]. Hyperplastic polyps have been shown to obstruct gastric emptying on rare occasions [6]. Any condition that may result in obstruction between the distal stomach and the distal duodenum has the potential to present with GOO. One uncommon etiology of GOO is due to the formation of a bezoar.

A bezoar is an amalgamation of indigestible material that most commonly forms within the stomach and infrequently forms within the duodenum. It can be attributed to surgical complications or altered gastric motility [7,8]. Bezoars occur with increased consumption of indigestible materials. The most common classifications of bezoars are phytobezoars, trichobezoars, pharmacobezoars, and lactobezoars, which are composed of fibrous vegetable material, hair, undigested medications/capsules, and milk curds, respectively [8,9].

The development of bezoars carries a prevalence of 0.4%, though specific incidence is dependent on the nature of the bezoar [8]. Certain risk factors increase a patient’s risk of developing bezoars, with prior gastric surgery and altered gastric motility being the most common. Gastric surgery may create an acidic environment with altered motility that predisposes to bezoar formation [10]. A prior case review revealed that bilateral truncal vagotomy with pyloroplasty was the most common surgery to result in bezoar formation [11]. Any etiology of gastroparesis can also result in bezoar formation.

Bezoars can be asymptomatic until they reach a size that causes significant luminal obstruction. These obstructions most commonly occur at the pylorus, as bezoars tend to form in the stomach. Patients then present with classic symptoms of GOO, including nausea and vomiting, upper abdominal pain, and anorexia [12]. It’s not uncommon for the patient to have secondary anemia and leukocytosis due to related GI bleeding, malabsorption, and/or inflammation. On a physical exam, the patient may present with a movable abdominal mass, depending on the size of the bezoar [9,13]. The following case is a presentation of a man diagnosed with GOO secondary to a phytobezoar that was endoscopically removed.

## Case presentation

**Figure 1:**
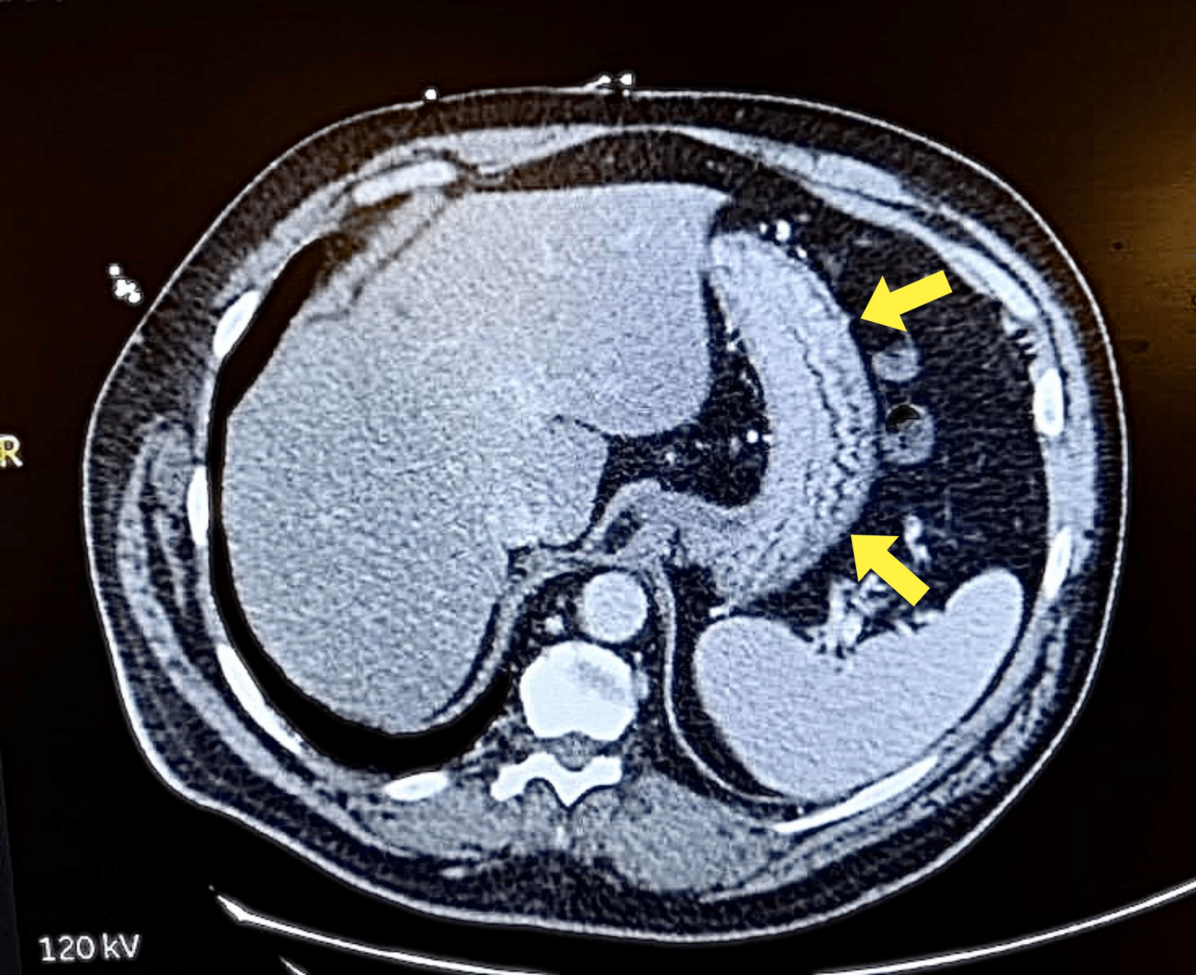
Transverse contrast CT abdomen showing gastric dilation

A 47-year-old male presented with a significant 15-pound unintentional weight loss, chronic intermittent nausea, and right upper abdominal pain for six weeks. His symptoms, which were particularly severe in the morning and worsened with eating, were managed with ondansetron and pantoprazole. The patient denied any non-steroidal anti-inflammatory drugs (NSAID) use and had no history of peptic ulcer disease. Notably, he underwent a cholecystectomy 10 years ago. When obtaining a dietary history, the patient admitted to consuming a diet high in fibrous vegetables. Upon physical examination, there was mild tenderness in the right upper quadrant without any palpable masses. An abdominal succussion splash was present. Laboratory tests, including complete blood count (CBC), comprehensive metabolic panel (CMP), urinalysis, and liver enzymes, were within normal limits, indicating no immediate hepatobiliary concerns. A contrast CT scan revealed gastric dilation, raising suspicion of gastric outlet obstruction (Figure 1).

**Figure 2:**
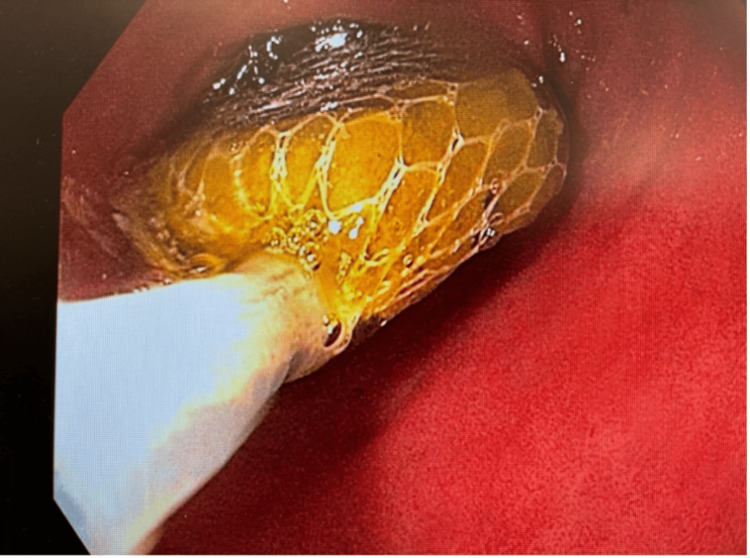
Endoscopic visualization of phytobezoar in the first part of the duodenum

Upper endoscopy revealed a phytobezoar in the first part of the duodenum (Figure 2). No associated ulcers or strictures were observed, which might have suggested a more complex underlying pathology. The phytobezoar was successfully removed endoscopically, resulting in the resolution of the patient's symptoms within 48 hours. To rule out any underlying motility disorders or anatomical abnormalities, a small bowel follow-through was performed and yielded normal results. The patient was advised on dietary modifications to prevent future bezoar formation and scheduled for a follow-up to monitor his recovery and ensure no recurrence of symptoms.

## Discussion

GOO is a clinical syndrome caused by a blockage in the gastrointestinal tract near the distal stomach or within the duodenum. GOO classically presents with anorexia, upper abdominal pain, and nausea and vomiting, regardless of its etiology. It is most commonly caused by malignancy, with the incidence being more than 50% [14]. Benign causes include PUD, iatrogenesis, and bezoars on occasion.

Bezoars are foreign concretions of indigestible material that form due to gastroparesis and are frequently located within the stomach. They are rare complications that have a prevalence of less than 1% and are classified by their composition [8]. Phytobezoars are formed of fibrous vegetable material, trichobezoars contain hair, pharmacobezoars are formed from undigested pill products, and lactobezoars are composed of milk curds. Prior literature suggests that a low-acid environment can predispose to bezoar formation, such as chronic use of proton pump inhibitors [10]. We do not believe that this was the catalyst for bezoar formation in our patient, as he began taking pantoprazole to manage his symptoms caused by the phytobezoar.

In addition to the classical presentations of GOO, gastric bezoars may also present with a movable abdominal mass, mild secondary anemia, and mild leukocytosis [9,12]. Plain abdominal radiography is the preferred means of diagnosing GOO caused by a bezoar due to it being noninvasive. Computer tomography and endoscopy have been used as diagnostic measures as well [11,15]. A CT scan was chosen for this patient due to the severity of his presentation.

Treatment of GOO revolves around prompt removal of the offending agent. This includes resection and treating malignancy, curing *H. pylori* infection, removing ulcer-inducing medications, or removal of foreign objects [1]. Benign GOO can be treated endoscopically with balloon dilation, with surgery reserved as a last resort [2].

Treatment options for gastric bezoars include fragmentation and milking, surgical removal, dissolution, and prokinesis. Preoperative endoscopy should be done in patients with symptomatic bezoars. Fragmentation of bezoars can be done endoscopically or enzymatically [11]. Gastric bezoars are milked into the small bowel after fragmentation, and intestinal bezoars are milked into the cecum [11]. Surgical treatment of bezoars includes endoscopic removal, which carries a success rate of 30.7% for trichobezoars [16]. Dissolution via nasogastric lavage with cola soda has been shown to be effective for gastric phytobezoar treatment [15,17,18]. Dissolution can also be achieved with cellulase or papain [19]. In a study of 52 patients with phytobezoars, the dissolution of phytobezoars was successful in 91% of cases, leaving it as a minimally invasive option for phytobezoar treatment [20]. For the presented case, the patient was treated with endoscopic removal of the phytobezoar without complications and underwent a small bowel follow-through with normal results.

## Conclusions

Phytobezoars are rarely found in the duodenum, as in the case of this patient. With the absence of motility abnormalities, the cause was determined to be dietary in origin, and the patient was counseled on nutritional modifications to prevent future bezoar formation. The patient had complete resolution of symptoms following endoscopic removal of the phytobezoar. For such a rare etiology of duodenal GOO, there are many treatment options at clinicians’ disposal for bezoar removal. It is important to be aware of all etiologies of GOO and the treatment modalities available.
